# Cell-free fat extract attenuates osteoarthritis via chondrocytes regeneration and macrophages immunomodulation

**DOI:** 10.1186/s13287-022-02813-3

**Published:** 2022-04-01

**Authors:** Zhuoxuan Jia, Bijun Kang, Yizuo Cai, Chingyu Chen, Zheyuan Yu, Wei Li, Wenjie Zhang

**Affiliations:** Department of Plastic and Reconstructive Surgery, Shanghai 9th People’s Hospital, Shanghai Jiao Tong University School of Medicine, Shanghai Key Laboratory of Tissue Engineering, National Tissue Engineering Center of China, 639 ZhiZaoJu Road, Shanghai, 200011 China

**Keywords:** Osteoarthritis, Cartilage regeneration, Synovitis, Macrophage

## Abstract

**Background:**

The prevalence of osteoarthritis (OA) is increasing, yet clinically effective and economical treatments are unavailable. We have previously proposed a cell-free fat extract (CEFFE) containing multiple cytokines, which possessed antiapoptotic, anti-oxidative, and proliferation promotion functions, as a “cell-free” strategy. In this study, we aimed to evaluate the therapeutic effect of CEFFE in vivo and in vitro.

**Methods:**

In vivo study, sodium iodoacetate-induced OA rats were treated with CEFFE by intra-articular injections for 8 weeks. Behavioral experiments were performed every two weeks. Histological analyses, anti-type II collagen, and toluidine staining provided structural evaluation. Macrophage infiltration was assessed by anti-CD68 and anti-CD206 staining. In vitro study, the effect of CEFFE on macrophage polarization and secretory factors was evaluated by flow cytometry, immunofluorescence, and quantitative reverse-transcription polymerase chain reaction (qRT-PCR). The effect of CEFFE on cartilage regeneration was accessed by cell counting kit-8 assay and qRT-PCR. The generation of reactive oxygen species (ROS) and levels of ROS-related enzymes were investigated by qRT-PCR and western blotting.

**Results:**

In rat models with sodium iodoacetate (MIA)-induced OA, CEFFE increased claw retraction pressure while decreasing bipedal pressure in a dose-dependent manner. Moreover, CEFFE promoted cartilage structure restoration and increased the proportion of CD206^+^ macrophages in the synovium. In vitro, CEFFE decreased the proportion of CD86^+^ cells and reduced the expression of pro-inflammatory factors in LPS + IFN-γ induced Raw 264.7. In addition, CEFFE decreased the expression of interleukin-6 and ADAMTs-5 and promoted the expression of SOX-9 in mouse primary chondrocytes. Besides, CEFFE reduced the intracellular levels of reactive oxygen species in both in vitro models through regulating ROS-related enzymes.

**Conclusions:**

CEFFE inhibits the progression of OA by promoting cartilage regeneration and limiting low-grade joint inflammation.

**Graphical abstract:**

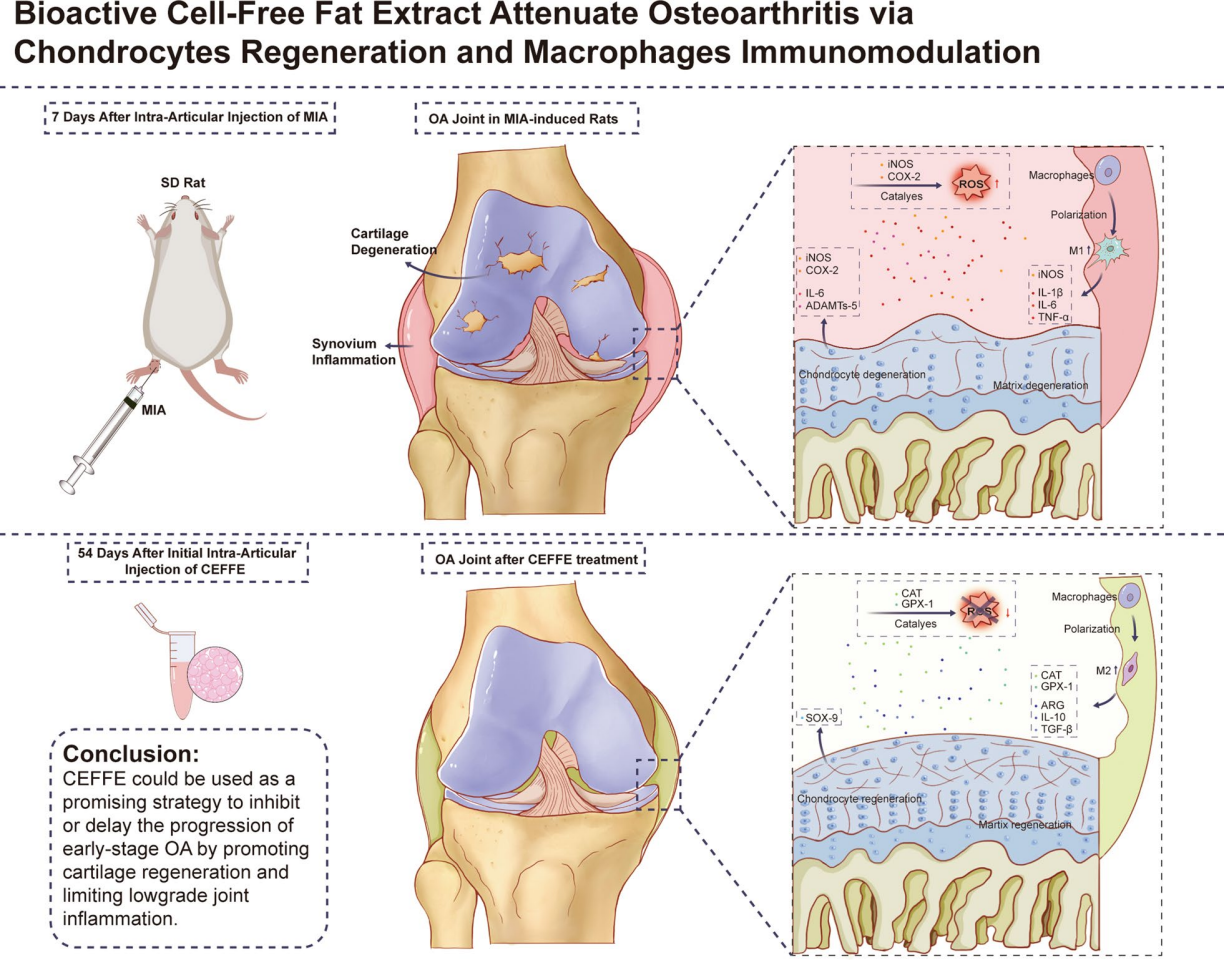

**Supplementary Information:**

The online version contains supplementary material available at 10.1186/s13287-022-02813-3.

## Background

Osteoarthritis (OA) is a common degenerative disorder characterized by the gradual degradation of articular cartilage, synovial inflammation, and remodeling of the subchondral bone [1, 2]. The physiological symptoms of OA mainly include pain, joint stiffness, and reduced motion, all of which significantly affect the quality of life of the patient [3–5]. However, current therapies for OA include nonsurgical management (e.g., weight reduction, exercises) and pharmacological interventions such as nonsteroidal anti-inflammatory drugs and paracetamol, which only temporarily alleviate clinical symptoms rather than repairing the tissues lesions or inhibiting the progression of OA [6–8]. Total joint arthroplasty is an effective treatment for the end-stage joint disease; however, the longevity of the prosthetic is limited, and the functional outcomes might be poor [9, 10]. Hence, in our study, we shifted the focus towards the treatment of early OA [9].

Although the pathological mechanisms are not well elucidated, accumulating evidence has suggested that apoptosis of joint cartilage tissues and the activation of innate inflammatory pathways (synovial macrophages in particular) play a critical role in the development of OA [11, 12]. Specifically, long-term cartilage degradation results in a release of tissue and molecular fragments as damage-associated molecular patterns that in turn activate synovial macrophages for the removal of these fragments [13]. However, synovial macrophages are over-activated to further damage the cartilage and form a positive feedback [7, 13]. This phenomenon is closely related to the subtypes of macrophages, including the pro-inflammatory M1 or anti-inflammatory M2 states [14]. M1 macrophages secrete many pro-inflammatory cytokines and mediators, such as tumor necrosis factor-α (TNF-α) and interleukin (IL)-1β, that recruit other immune cells to phagocyte cell debris [15, 16]. In contrast, M2 macrophages secrete anti-inflammatory factors such as IL-10 and overexpress the mannose receptor (CD206), promoting tissue repair [15, 16] Meanwhile, the imbalance between the production of reactive oxygen species (ROS) containing free radicals such as hydrogen peroxide, hydroxyl radical, superoxide anion, and nitric oxide and their clearance by the antioxidant defense system including various enzymes, such as catalases (CAT), glutathione peroxidase (GPX), and superoxide dismutase (SODs), is a significant cause of chronic inflammation [17–19]. Therefore, due to the presence and interactions of these inflammatory mediators and oxidative stress, the joint cavity remains in an inflammatory state for a long time, which accelerates the progress of cartilage degradation and joint dysfunction [17, 20].

As a result, the key to the treatment of OA is to develop a multifunctional agent with anti-inflammatory properties that can change the pro-inflammatory microenvironment and promote cartilage regeneration [14, 21]. Clinical trials and basic research studies recently have revealed the therapeutic properties of mesenchymal stem cells (MSCs), skeletal stem cells, and adipose-derived stem cells (ADSCs), which could be applied in cell therapy for OA [22–27]. In addition, accumulating evidence has demonstrated that several stem cells mainly exert their regenerative and immunomodulatory effects through a paracrine manner by releasing abundant growth factors and anti-inflammatory cytokines [22–25]. Of these, ADSCs represent valid candidates attributed to their anti-inflammatory and chondroprotective effects as MSCs and their simple acquisition from liposuction wastes [25, 27].

Although ADSCs have numerous advantages, immune rejection and the tumorigenicity of cell-based therapies have restricted their application [28–30]. To overcome these restrictions and facilitate the use of adipose tissue in treating OA, cell-free fat extract (CEFFE), the liquid fraction isolated from liposuction wastes was generated [31]. According to our previous study, CEFFE is a kind of cell-free liquid that is easily obtained using a mechanical approach to remove cellular components and lipid residues [31]. CEFFE is rich in various growth factors and anti-inflammatory components, including insulin-like factor-1 (IGF-1), transforming growth factor (TGF)-β, vascular endothelial growth factor (VEGF), and basic fibroblast growth factor, which is similar to the paracrine factors of ADSCs [31–37]. In addition, our previous studies have demonstrated that CEFFE has antiapoptotic, anti-oxidative, and proliferation promotion abilities [31–38]. Based on these findings, we hypothesized that CEFFE might have a therapeutic effect on early OA. To this end, our present study evaluated the effects of CEFFE on cartilage regeneration and macrophage polarization and explored its potential mechanisms.

## Materials and methods

### Preparation of CEFFE

**Fig. 1:**
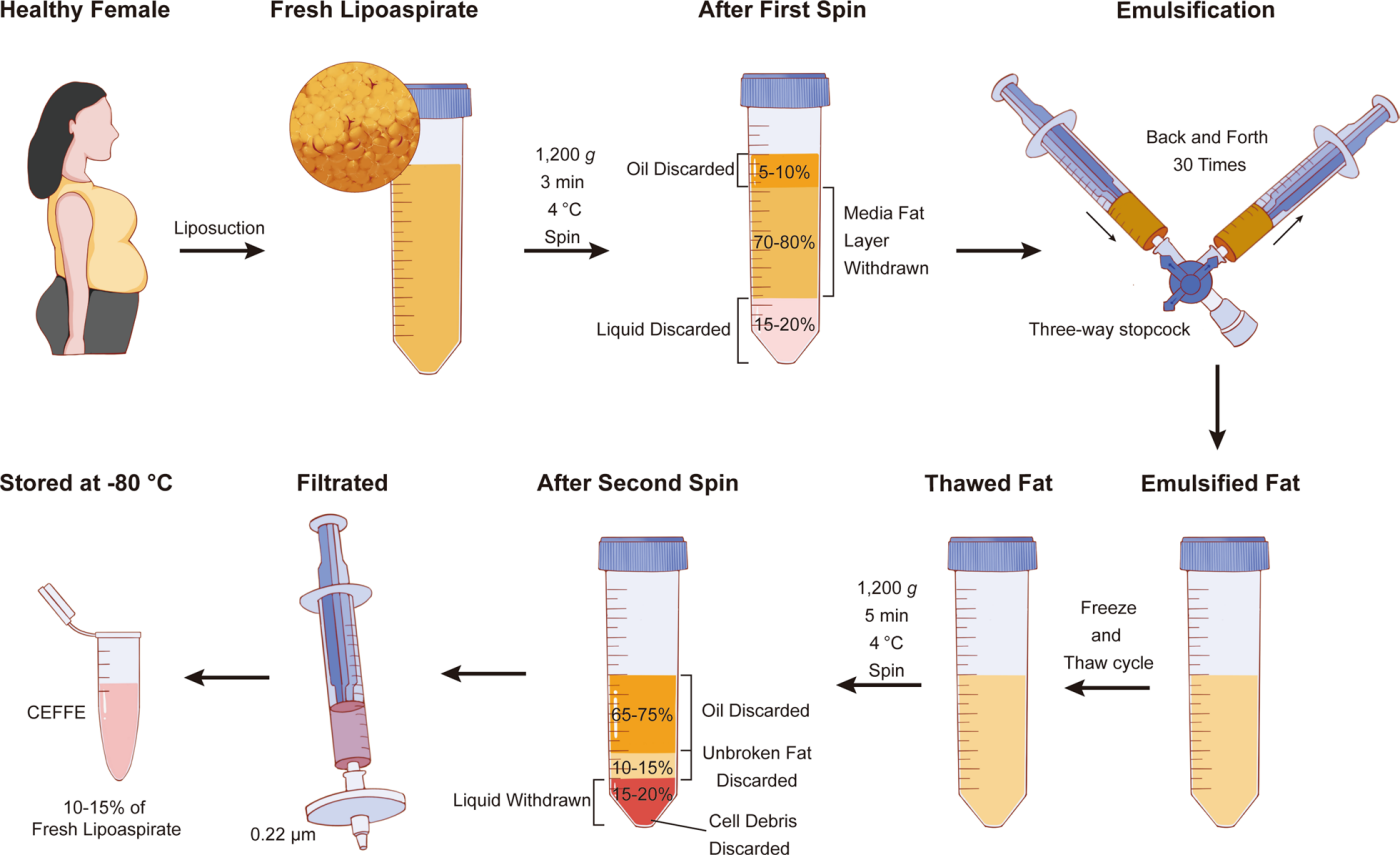
Schematic illustration of the preparation of CEFFE

The Ethics Committee of Shanghai Ninth People's Hospital approved all experimental protocols of the present study. After informed consent was obtained, adipose tissue was harvested from the abdomen or thigh of healthy adult females using liposuction from December 2020 to November 2021. CEFFE used in this study is a mixture derived from 5 healthy female participants aged 22–35. The preparation of CEFFE was carried out following a previously established method [31] (Fig. 1). In brief, adipose tissues were rinsed with physiological saline solution (Kelun Pharmaceutical Co., Ltd, Sichuan, China) to wash away blood and tissue debris. Following centrifugation at 1200 g for 3 min, three layers were formed. The upper oil layer and the lower aqueous layer were removed, whereas the middle fat layer was retained for mechanical emulsification by shifting between two 10-mL syringes (KDL, Zhejiang, China), which were attached to a three-way stopcock with an internal diameter of 2 mm (Terumo Corporation, Tokyo, Japan), 30 times. The emulsified fat was then frozen and stored at -80 °C and thawed rapidly at 37 °C to break the cell membranes. After one freeze–thaw cycle, samples were centrifuged at 1200 g for 5 min, resulting in the formation of four layers. Finally, the third liquid layer was collected and filtered through a 0.22-µm filtration membrane (Corning Glass Works, Corning, NY, USA) to remove bacteria and other debris, generating CEFFE, which was frozen at − 80 °C for further experiments. The protein concentration in CEFFE was determined using a bicinchoninic acid assay kit (BCA; Sigma-Aldrich, St Louis, MO, USA).

### Animals

**Fig. 2:**
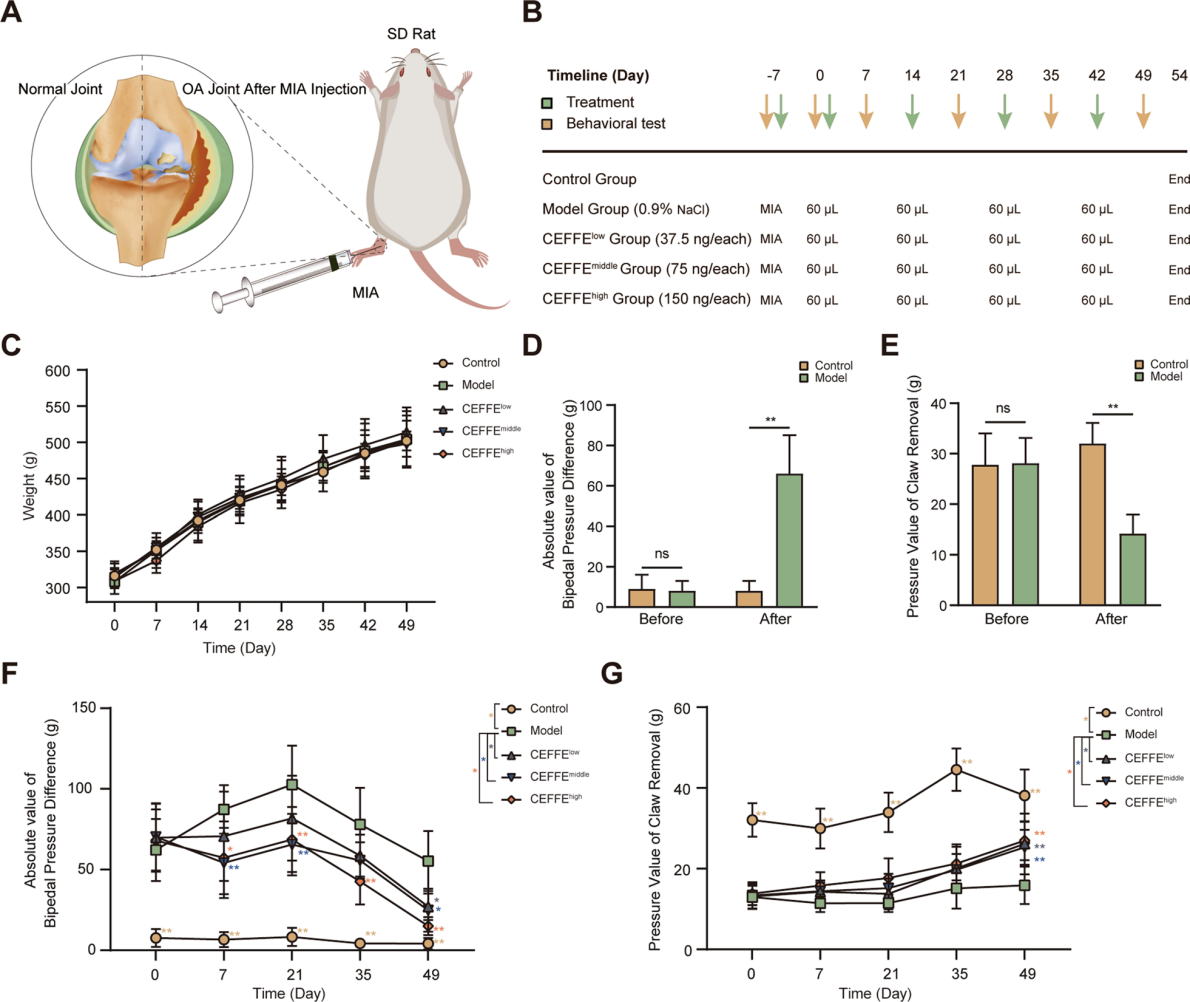
Therapeutic efficacy of CEFFE on the symptoms of MIA-induced OA rats. **A** Schematic overview of MIA intra-articular injection. **B** Flowchart of the dosing regimen and behavioral experiments. **C** No statistical differences in body weight changes among different groups at each time point. **D** Changes in absolute bipedal pressure difference after injection of MIA. **E** Changes in claw retraction pressure after injection of MIA. **F** Quantitative analysis of absolute bipedal pressure difference after injection of CEFFE at each time point. **G** Quantitative analysis of claw retraction pressure after injection of CEFFE at each time point**.** Data represent the mean ± SD (*n* = 6 per group). *p < 0.05; **p < 0.01; ns, no significant difference between groups

Adult male Sprague–Dawley (SD) rats (7–8 weeks old, 290–344 g) were purchased from Vital River Laboratory Animal Technology Co., Ltd (SCXK-2016–0006, Beijing, China). All rats were housed in a barrier environment (temperature, 20–25 °C; humidity, 40–70%; light, 12 h/d) with free access to water and food (SYXK-2019-0013). All experiments were approved by the Animal Care and Experiment Committee of Shanghai Jiao Tong University School of Medicine. Rats were randomly divided into 5 groups (*n* = 6 in each group): control, model, CEFFE^low^, CEFFE^middle^, and CEFFE^high^. Rats in the control group were not subjected to any treatment from Day 7 to the end. A single intra-articular injection of 50 µL sodium iodoacetate (MIA, 40 mg/mL dissolved in saline; Sigma-Aldrich, St Louis, MO, USA) was administered with a 30-gauge needle to rats in other groups to induce the OA model. On Day 0, 14, 28, and 42, MIA-injected rats were then given multiple 60 µL intra-articular injection of saline, CEFFE^low^ (15 µL CEFFE + 45 µL saline, 0.0357 mg/knee), CEFFE^middle^ (30 µL CEFFE + 30 µL saline, 0.075 mg/knee), CEFFE^high^ (60 µL CEFFE, 0.15 mg/knee) with a 30-gauge needle four times in total (Fig. 2A, B). Measurement of bipedal balance using weight-bearing asymmetry [39] and von Frey test using the dynamic plantar tactile device [40] were performed on all surviving animals on Day—7, 0, 7, 21, 35, and 49. All animals were euthanized on Day 54.

### Histological and immunohistochemistry

The entire left knee joint of rats was removed, fixed in 10% buffered formaldehyde, and decalcified in 10% formic acid. After embedding, the knee joint medial compartment was cut into 5-µm-thick sagittal oriented sections. Sections were stained with hematoxylin–eosin (HE) and Safranin O-fast green (S&F; Solarbio, Beijing, China) to observe the cartilage structure. Three visual fields were randomly selected for the evaluation of HE structural scoring using a 1–4 grading system (1 = slight; 2 = mild; 3 = moderate; 4 = severe). For S&F structural scoring, the Osteoarthritis Research Society International (OARSI) joint pathology scoring standards were referred to [41]. To observe glycosaminoglycan (GAG), sections were stained with toluidine blue (Solarbio, Beijing, China).

To evaluate the regeneration and maturation of cartilage, immunohistochemistry for type II collagen was performed. To assess macrophage infiltration in the synovium of the joint, immunohistochemistry for CD68 and CD206 was carried out. To assess angiogenic properties of CEFFE in the synovium of the joint, immunohistochemistry for CD31 was carried out. Briefly, sections were dewaxed and hydrated with dimethyl benzene and a graded ethanol series. Next, sections were placed in a repair box filled with citric acid antigen repair buffer (pH = 6.0, Beyotime, China), boiling for 1 h to repair antigen. 0.2% Triton (Triton X-100, Solarbio, Beijing, China) was used to permeabilize tissues, and 3% H_2_O_2_ (Yaji Biological, Shanghai, China) was used to block endogenous peroxidase. Then, sections were blocked with goat serum (Solarbio, Beijing, China) for 1 h and incubated with antibodies to type II collagen (1:300, Abcam, Cambridge, UK), CD68 (1:200, Abcam, Cambridge, UK), CD206 (1:200, Abcam, Cambridge, UK), CD31 (1:200, Abcam, Cambridge, UK) at 4 ℃ overnight, respectively. Finally, sections were incubated with HRP-labeled secondary antibody (Beyotime, China) for 30 min at 37 ℃ and visualized with a 3, 3′-diaminobenzidine kit (DAB Substrate Kit, Burlingame, CA, USA), followed by counterstaining with hematoxylin. Three randomly selected fields from each section were imaged with light microscopy (Nikon Eclipse 90i, Japan). The number of CD68-, CD206-positive cells, and CD31-positive capillaries were calculated using Image-Pro Plus 6 software (Rockville, MD, USA).

### Cell culture

To investigate the role of CEFFE in regulating inflammation in vitro, mouse macrophage Raw 264.7 cells were purchased from the American Type Culture Collection (ATCC) cell bank (ATCC Number: TIB-71™, Shanghai, China). Raw 264.7 cells were cultured in Dulbecco’s modified Eagle medium (DMEM; Invitrogen, Thermo Fisher Scientific, USA) supplemented with 10% fetal bovine serum (FBS; Gibco, Gland Island, NY, USA) and 1% penicillin–streptomycin-gentamicin solution (Gibco, Gland Island, NY, USA) and maintained at 37 °C in a humidified 5% CO_2_ atmosphere.

### In vitro polarization induction of Raw 264.7 macrophages

To investigate the role of CEFFE in regulating macrophage polarization in an inflammatory environment, 4 × 10^5^ Raw 264.7 cells were seeded in 6-well plates. After 24 h of cell contact, the normal medium was replaced with 2 mL culture medium (control group); culture medium containing 1 µg/mL lipopolysaccharide (LPS; Sigma-Aldrich, St. Louis, MO, USA) and 30 ng/mL interferon-γ (IFN-γ; PeproTech, Rocky Hill, USA) (LPS + IFN-γ group); culture medium containing 1 µg/mL LPS, 30 ng/mL IFN-γ; and different concentrations of CEFFE (100 µg/mL, 250 µg/mL, and 500 µg/mL). After incubating in these media for 24 h, all media were replaced with fresh media for another 24 h. The polarization of Raw 264.7 cells to M1/M2 macrophages was identified by flow cytometry, immunofluorescence staining, and quantitative real-time polymerase chain reaction (qRT-PCR). We also tested the effects of different concentrations of CEFFE on inactive macrophages in the same way.

### Flow cytometry

For cytometry, treated cells were blown down using cold phosphate-buffered saline (PBS, Invitrogen, San Diego, CA, USA), and 4 × 10^5^ cells were counted and suspended in 100 µL PBS supplemented with 4% FBS. Cells were then incubated with fluorescein isothiocyanate-anti-mouse CD86 (1:40; BioLegend, San Diego, CA, USA), allophycocyanin-anti-mouse CD206 (1:40; BD Pharmingen™, San Diego, CA, USA) at 4 °C for 30 min. After washing thrice with PBS, labeled cells were suspended in 100 µL PBS, and data were acquired via the fluorescence-activated cell sorting Calibur flow cytometry system (BD Bioscience, San Jose, CA, USA) and analyzed using the CytExpert software (Beckman Coulter, Inc., Brea, CA, USA).

### Immunofluorescence staining

For immunofluorescence staining, RAW 264.7 cells were seeded into 6-well plates with cell climbing slices at a density of 2 × 10^5^ cells/well. As previously described, after 48 h of treatment, cells were fixed with 4% paraformaldehyde in PBS and incubated with anti-CD86 (1:200; ProteinTech, Wuhan, China) overnight at 4 °C followed by 1-h incubation with Alexa Fluor 488-conjugated goat secondary antibody (Jackson ImmunoResearch, Carlsbad, CA, USA) at room temperature [42]. Nuclei were stained with DAPI (1:1000, Boster, Wuhan, China).

### Quantitative real-time PCR

To determine the levels of expression of the M1-related paracrine factors [IL-1β, IL-6, inducible nitric oxide synthase (iNOS), and TNF-α], M2-related factors [IL-10, arginase-1 (ARG), TGF-β, and CD-206], and oxidative stress-related enzymes [GPX-1, CAT, SOD-1, and SOD-2], treated cellular mRNA was extracted using the Total RNA Extraction Reagent (EZBioscience, Roseville, USA) according to the manufacturer's instructions. Briefly, 1 µg total RNA was reverse transcribed to cDNA using a reverse transcription master mix (EZBioscience, Roseville, USA) according to the manufacturer's instructions. Subsequently, qRT-PCR was conducted using an SYBR Green qPCR master mix (ROX2 plus; EZBioscience, Roseville, USA). Cycling parameters were 95 °C for 5 min, then 40 cycles at 95 °C for 10 s followed by 60 °C for 30 s. At least three technical replicates were performed for each sample. Relative expression levels were calculated using the 2^−ΔΔCq^ method and are presented as fold-change relative to the glyceraldehyde 3-phosphate dehydrogenase house gene expression [43]. Primers for qRT-PCR are listed in Additional file 2: Table S1.

### Measurement of reactive oxygen species

The levels of intracellular ROS were assessed using a ROS assay kit (Beyotime, China) according to the manufacturer's instructions. Briefly, cells were washed thrice with non-FBS DMEM and incubated with 10 µM 2,7-dichlorodihydrofluorescein diacetate (DCFH-DA) at 37 °C for 20 min in the dark. After washing thrice with non-FBS DMEM, cells were observed and imaged under a fluorescence microscope (Olympus Corporation, Tokyo, Japan). Cells were digested and suspended in 100 µL PBS supplemented with 4% FBS to detect ROS levels using flow cytometry (Beckman-Coulter, Brea, CA, USA).

### Levels of nitric oxide

Nitrite accumulated in the culture supernatant was measured as an indicator of the production of NO using a NO assay kit (Beyotime, China) according to the manufacturer's instructions. In brief, 50 µL of culture supernatant or NaNO_2_ standard was mixed with 100 µL Griess reagent at 25 °C. After incubation, absorbance was read at a wavelength of 540 nm using a microplate reader (SpectraMAX190; Molecular Devices, Sunnyvale, CA), and NO concentrations were estimated from the NaNO_2_ standard curve.

### Western blotting

Cells were cultured in a 6-well plate. After treatment, the total protein of cells was obtained and quantified using a BCA assay (Sigma-Aldrich, St Louis, MO, USA). Proteins were separated according to their molecular weights through sodium dodecyl sulfate–polyacrylamide gel electrophoresis and transferred to polyvinylidene fluoride membranes (Millipore, MA, USA). After successive incubation with primary and rabbit secondary antibody conjugated with HRP (Abcam, Cambridge, UK), membranes were observed using enhanced chemiluminescence (Pierce, Rockford, IL, USA). Primary antibodies used were anti-GPX-1, anti-CAT, anti-SOD-1, anti-SOD-2, anti-iNOS, anti-COX-2, and anti-β-actin (1:1000; Cell Signaling Technology, Inc., Danvers, MA, USA).

### Isolation and culture of mouse primary chondrocytes

Primary chondrocytes were obtained from male C57BL/6 mice aged 4 weeks. The femoral head was removed and cut into pieces, digested with trypsin for 40 min and then digested with type II collagenase for 8 h. The isolated chondrocytes were confirmed by toluidine staining (Solarbio, Beijing, China) and western blot of type II collagen (1:200, Abcam, Cambridge, UK) (Additional file 1: Fig. S1). Primary chondrocytes were cultured at F-12/DMEM (Invitrogen, Thermo Fisher Scientific, USA) supplemented with 10% FBS and 1% penicillin–streptomycin–gentamicin solution. Then, 10 nM IL-1β and 10 nM TNF-α (PeproTech Inc., Rocky Hill, NJ, USA) were used to induce inflammation in cultured primary chondrocytes.

### Cell counting kit-8 assay

Primary chondrocytes were cultured in a 96-well plate at 3 × 10^3^ per well until attached. After treatment with CEFFE with or without inflammation factors for 3 d, cells were incubated with DMEM supplemented with 10% Cell Counting Kit-8 reagent (Beyotime, China) at 37 °C for 2 h in the dark. The optical density of each sample was detected at a wavelength of 450 nm using a microplate reader (Thermo Electron Corporation, USA).

### Statistical analysis

Statistical analysis was performed using the IBM SPSS ® 24.0 statistical software (IBM Corporation, Chicago, IL, USA). Differences between groups were analyzed using one-way analysis of variance, followed by Tukey's post hoc test. Data are expressed as the mean ± standard deviation (SD). Statistical significance was indicated as *p* < 0.05* or *p* < 0.01**.

## Results

### CEFFE reduced the severity of symptoms in MIA-induced osteoarthritis rats

To evaluate the therapeutic value of CEFFE, we established a rat model of OA and examined the effect of the treatment with CEFFE on the injured articular cartilage. We did not observe any obvious clinical signs or weight differences among groups within the experiment duration (Fig. 2C). We also found that before modeling, there was no significant difference in bipedal pressure (28.1 ± 5 vs. 27.8 ± 6.2 g, *p* > 0.05) and claw retraction pressure (8 ± 5 vs. 9 ± 7, *p* > 0.05) between the control and model groups. However, we noticed a clear difference 7 days after modeling, suggesting that MIA can successfully induce OA in SD rats (Fig. 2D, E). More specifically, 7 days after the fourth administration, the absolute value of bipedal pressure of rats in the CEFFE^low^ group was lower than that in the model group. Moreover, we found that the absolute value of the CEFFE^middle^ and CEFFE^high^ groups was lower than that of the model group from the first administration until the end of the experiment (Fig. 2F). In particular, we observed that the claw retraction pressure of rats in the three CEFFE treatments groups was significantly higher than that in the model group at the end of the experiment (Fig. 2G). These results indicated that CEFFE could reduce the severity of symptoms in MIA-induced OA rats.

### Mechanisms of CEFFE-mediated cartilage repair in MIA-induced OA rats

**Fig. 3:**
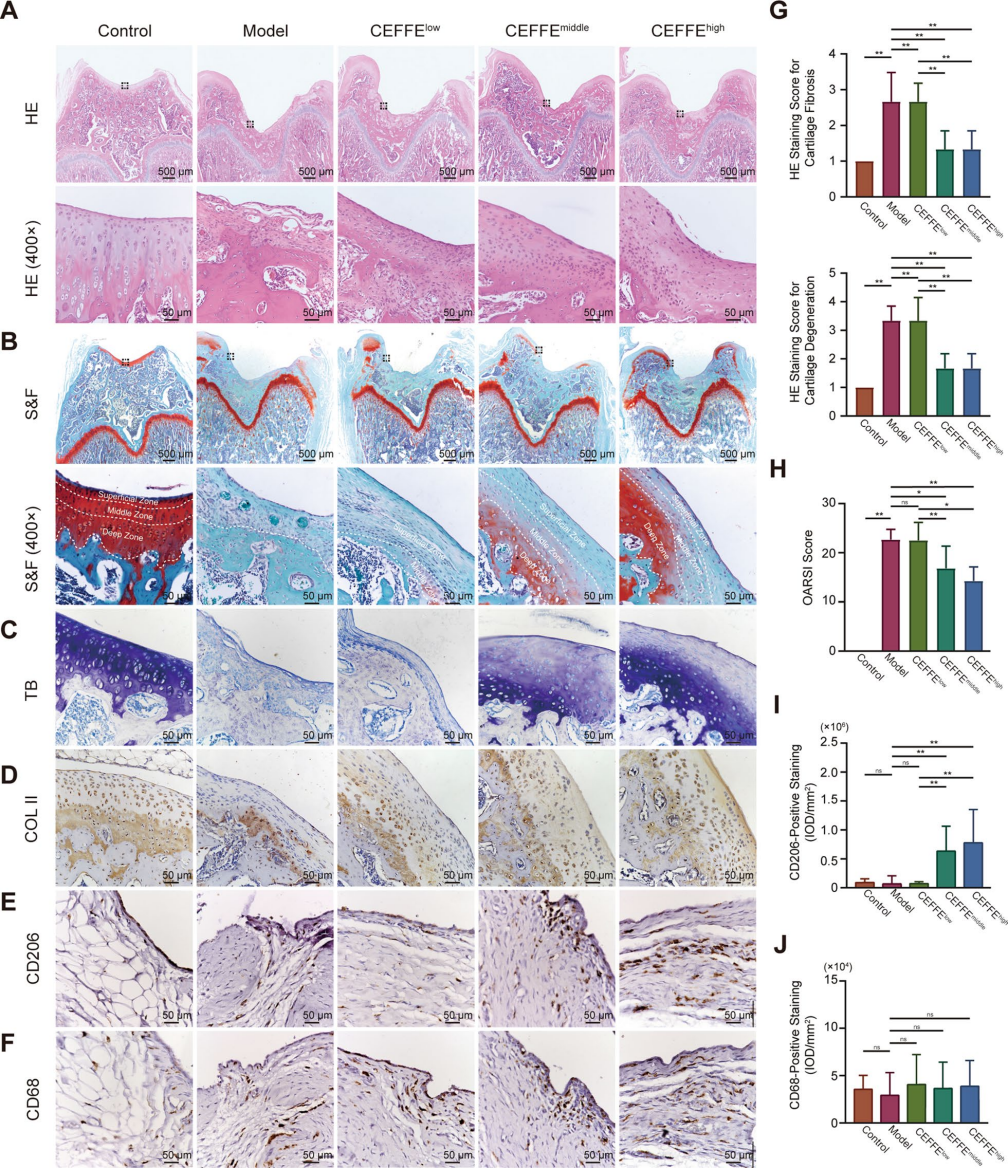
Histological evaluation of structural changes and matrix deposition in cartilage and macrophage infiltration in synovium. **A**, **G** The longitudinal section of a rat joint was stained with HE. Quantitative analysis of HE staining score containing cartilage fibrosis and cartilage degeneration according to a 1–4 grading system. **B**, **H** Safranin-O fast green (S&F) staining of the longitudinal section of a rat bone. Quantitative analysis of S&F staining according to the OARSI standard. **C** GAG staining of cortical bone sections. **D** Anti-COL II staining of cortical bone sections. **E**, **I** Anti-CD206 staining of knee synovium. **F**, **J** Anti-CD68 staining of knee synovium. Scale bars are noted on the right bottom corner of each picture. Data represent the mean ± SD (*n* = 6 per group). *p < 0.05; **p < 0.01; ns, no significant difference between groups

We conducted special staining and immunohistochemistry to investigate the mechanism of the CEFFE-mediated therapeutic effects. HE and S&F staining showed that compared with the model group, the gross morphologies of joint sections in the CEFFE^middle^ and CEFFE^high^ groups were closer to the control group. Specially, in the model group, the superficial zone was lost and the internal structure of articular cartilage was changed. In CEFFE treatment groups, the structure of cartilage was changed gradually to the normal control group as the CEFFE concentration increased (Fig. 3A, B). The score of HE staining (cartilage fibrosis and cartilage degeneration; Fig. 3G) and S&F staining (OARSI; Fig. 3H) also showed a decline trend as CEFFE concentration increased.

Moreover, toluidine blue staining and immunohistochemically detection of type II collagen revealed the reduction of both GAG and type II collagen in the cartilage matrix in the model group; however, this effect was reversed after intra-articular injection of CEFFE (Fig. 3C, D). According to the CD68 staining, we noticed that the infiltration of total macrophages (CD68-positive cells) was not different between the CEFFE-treated and model groups (Fig. 3F, G). However, we found that CEFFE upregulated the synovial M2 macrophage (CD206-positive cells) ratio compared with that in the model group (Fig. 3E, I). Furthermore, results of CD31 staining revealed an upward trend of capillary density in synovial tissue. However, only the CEFFE^middle^ group exhibited significant differences (*p* < 0.05) compared with Model group (Additional file 1: Fig. S2).

### CEFFE inhibited the transformation of Raw 264.7 cells from M0 to M1 macrophages

**Fig. 4:**
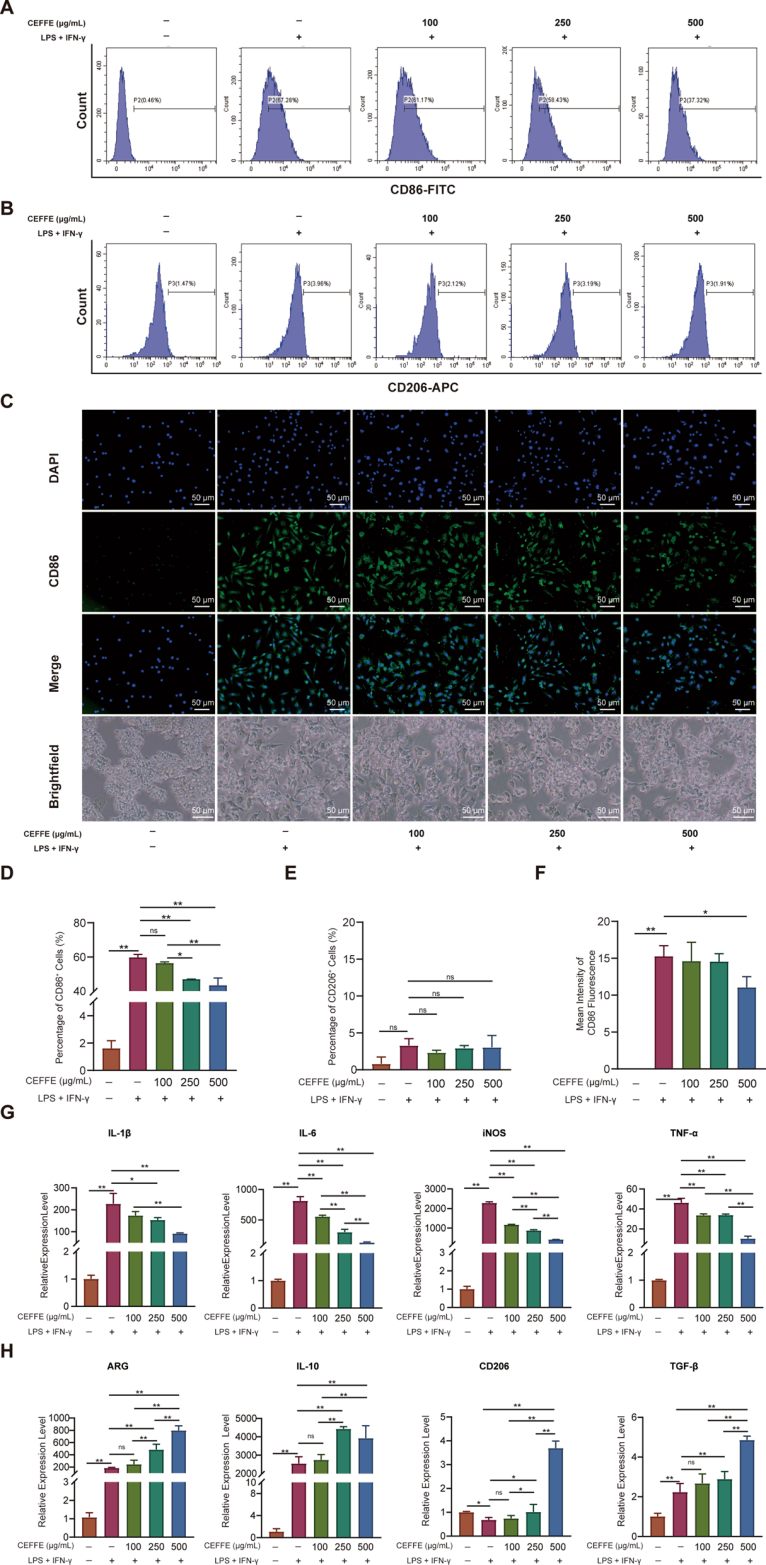
CEFFE attenuated the differentiation of Raw 264.7 cells from M0 to M1 macrophages. **A**, **D** Quantification of CD86-positive cells by flow cytometry. A significant decline in the proportion of M1 macrophages was observed after treatment with CEFFE. **B**, **E** Quantification of CD206 positive cells by flow cytometry. No obvious change was observed in the ratio of M2 cells. **C**, **F** Immunofluorescence staining of CD86 and bright-field pictures of Raw 264.7 cells. The CEFFE-treated group showed a declining tendency and morphological changes compared with the LPS + IFN-γ group. **G** Quantification of IL-1β, IL-6, iNOS, and TNF-α mRNA expression by qRT-PCR. The CEFFE-treated group showed a significant dose-dependent decrease compared with the LPS + IFN-γ group. **H** Quantification of ARG, IL-10, CD206, and TGF-β mRNA expression by qRT-PCR. The CEFFE-treated group showed a slight upward trend compared with the LPS + IFN-γ group. Scale bars are noted on the right bottom corner of each picture. Data represent the mean ± standard deviation (*n* = 3 per group). *p < 0.05; **p < 0.01; ns, no significant difference between groups

To verify that CEFFE exerted a beneficial effect in alleviating inflammation, we stimulated Raw 264.7 cells with LPS and IFN-γ and simultaneously incubated them with 100 µg/mL, 250 µg/mL, and 500 µg/mL CEFFE. Flow cytometry analysis indicated that LPS + IFN-γ significantly stimulated the polarization of M0 macrophages towards M1 (CD86-positive, pro-inflammatory cells) from 1.62 to 59.74%. However, we observed that the proportion of M1 macrophages (59.74 ± 1.69% to 56.9 ± 0.71%, 46.94 ± 0.05%, 46.43 ± 4.21%) was gradually decreased in a CEFFE concentration-dependent manner at 100 µg/mL, 250 µg/mL, and 500 µg/mL CEFFE, respectively (Fig. 4A, D). Meanwhile, we noticed that incubation of M0 cells with the same concentration of CEFFE did not lead to a marked change (Additional file 1: Fig. S3A, C).

Moreover, we detected a low proportion (1–13%) of M2 macrophages (CD206-positive, anti-inflammatory cells) when incubated with different concentrations of CEFFE with or without LPS + IFN-γ (Fig. 4B, E and Additional file 1: Fig. S3B, D). Our immunofluorescence analysis was consistent with that of flow cytometry. We detected an upward trend in the proportion of M1 cells (CD86 positive) in the LPS + IFN-γ group compared with that in the control group, whereas this trend was reversed after co-incubation with CEFFE (Fig. 4C, F).

To further explore the potential role of CEFFE in resolving inflammation, we conducted qRT-PCR for pro-inflammatory and anti-inflammatory factors. Our qRT-PCR analysis showed that the mRNA expression levels of the IL-1β, IL-6, iNOS, and TNF-α pro-inflammatory factors were remarkably elevated in the LPS + IFN-γ group. In contrast, we noticed that after co-incubation with different concentrations of CEFFE, the mRNA expression exhibited a significant dose-dependent decrease (Fig. 4G). Concurrently, we detected that the mRNA expression of the ARG, IL-10 and TGF-β anti-inflammatory factors showed a slight upward trend in the LPS + IFN-γ + CEFFE group when compared with that in the LPS + IFN-γ group (Fig. 4H).

### CEFFE promoted proliferation, regeneration, reduced matrix degradation, and reduced inflammation of murine primary chondrocytes

**Fig. 5:**
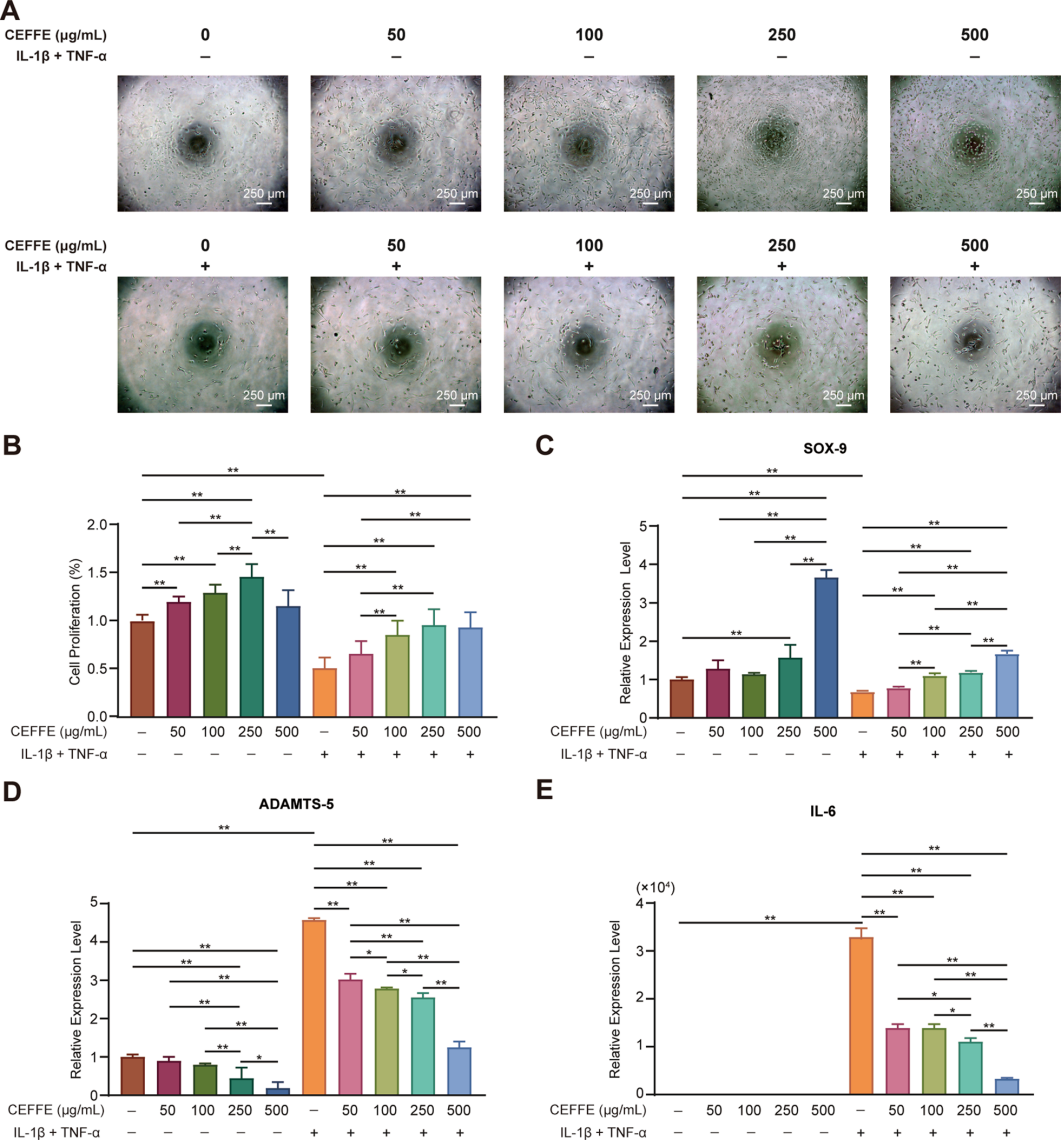
The protective effect of CEFFE in murine primary chondrocytes. **A**, **B** Original images and CCK8 results of murine primary chondrocytes treated with CEFFE, with or without IL-1β and TNF-α. CEFFE promoted the proliferation of murine primary chondrocytes regardless of IL-1β and TNF-α. **C** Quantification of SOX9 mRNA expression by qRT-PCR. **D** Quantification of ADAMTS-5 mRNA expression by qRT-PCR. **E** Quantification of IL-6 mRNA expression by qRT-PCR. Scale bars are noted on the right bottom corner of each picture. Data represent the mean ± SD (n = 3 per group). *p < 0.05; **p < 0.01; ns, no significant difference between groups

To investigate the effect of CEFFE on chondrocytes, we isolated and used murine primary chondrocytes. We treated cells with different concentrations of CEFFE (0, 50, 100, 250 and 500 µg/mL) in the presence or absence of IL-1β and TNF-α. We found that CEFFE promoted the proliferation of primary chondrocytes. Whereas inflammation factors distinctly inhibited the proliferation of cells, we noticed that CEFFE exerted a protective effect on the growth of primary chondrocytes under inflammation (Fig. 5A, B).

We further stimulated chondrocytes for increased matrix degradation and inhibition of chondrocyte regeneration under inflammation. To this end, we detected the expression of interleukin-6 (IL-6) as a pro-inflammation marker, a disintegrin metalloproteinase with thrombospondin motifs 5 (ADAMTs-5) as the most significant matrix degradation enzymatic marker, and SOX-9 as a chondrocyte regeneration marker. We found that the expression of SOX-9 was increased, whereas that of ADAMTs-5 was decreased when primary chondrocytes were treated with CEFFE. Moreover, we noticed that while primary chondrocytes were co-cultured with inflammation factors, the expression of SOX-9 was decreased, whereas that of ADAMTs-5 and IL-6 was increased. Furthermore, after treatment of chondrocytes under inflammation with CEFFE, we found that the expression of SOX-9 was increased, whereas that of ADAMTs-5 and IL-6 was decreased (Fig. 5C–E).

### CEFFE inhibited inflammation-induced oxidative stress in RAW264.7 macrophages and murine primary chondrocytes

**Fig. 6:**
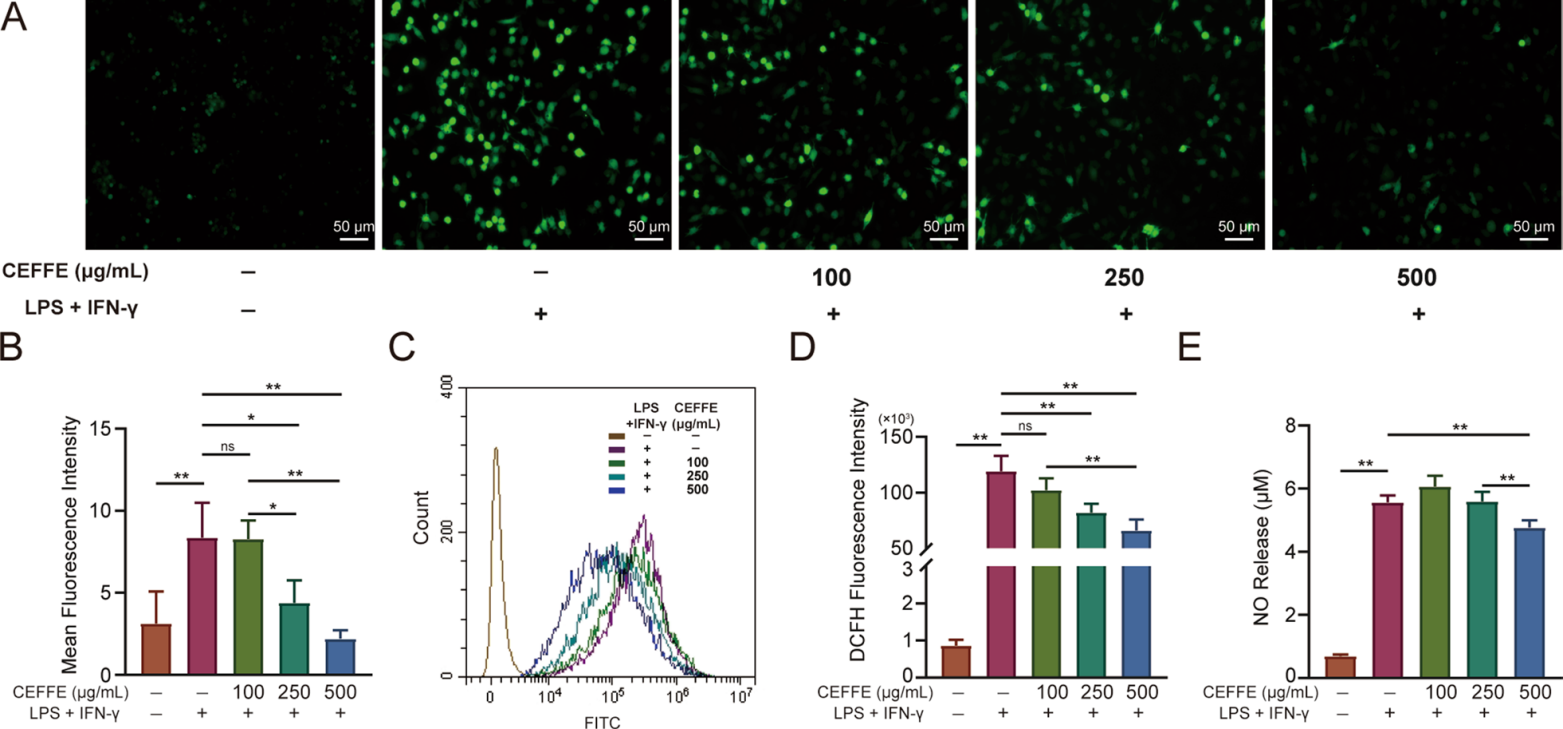
CEFFE reduced the intracellular production of ROS in RAW 264.7 cells. **A**, **B** Fluorescence microscopy observation and the qualification of mean fluorescence intensity. **C**, **D** Flow cytometry showed that CEFFE counteracted the intracellular production of ROS induced by LPS + IFN-γ in a dose-dependent manner. **E** Intracellular NO was measured using a nitric oxide assay kit. Scale bars are noted on the right bottom corner of each picture. Data represent the mean ± SD (*n* = 3 per group). *p < 0.05; **p < 0.01; ns, no significant difference between groups

To explore the mechanism of action of CEFFE on RAW264.7 macrophages and primary chondrocytes, we detected the intracellular levels of ROS through DCFH-DA staining. We observed the fluorescence intensities of RAW264.7 macrophages and primary chondrocytes using fluorescent microscopy and flow cytometry. We found that in RAW264.7 macrophages, CEFFE reduced the fluorescence intensity induced by LPS and IFN-γ in a dose-dependent manner (Fig. 6A–D). Concomitantly, we noticed that nitrite accumulation in the culture supernatant decreased after incubation with 500 µg/mL CEFFE compared to the LPS + IFN-γ group (Fig. 6E).

**Fig. 7:**
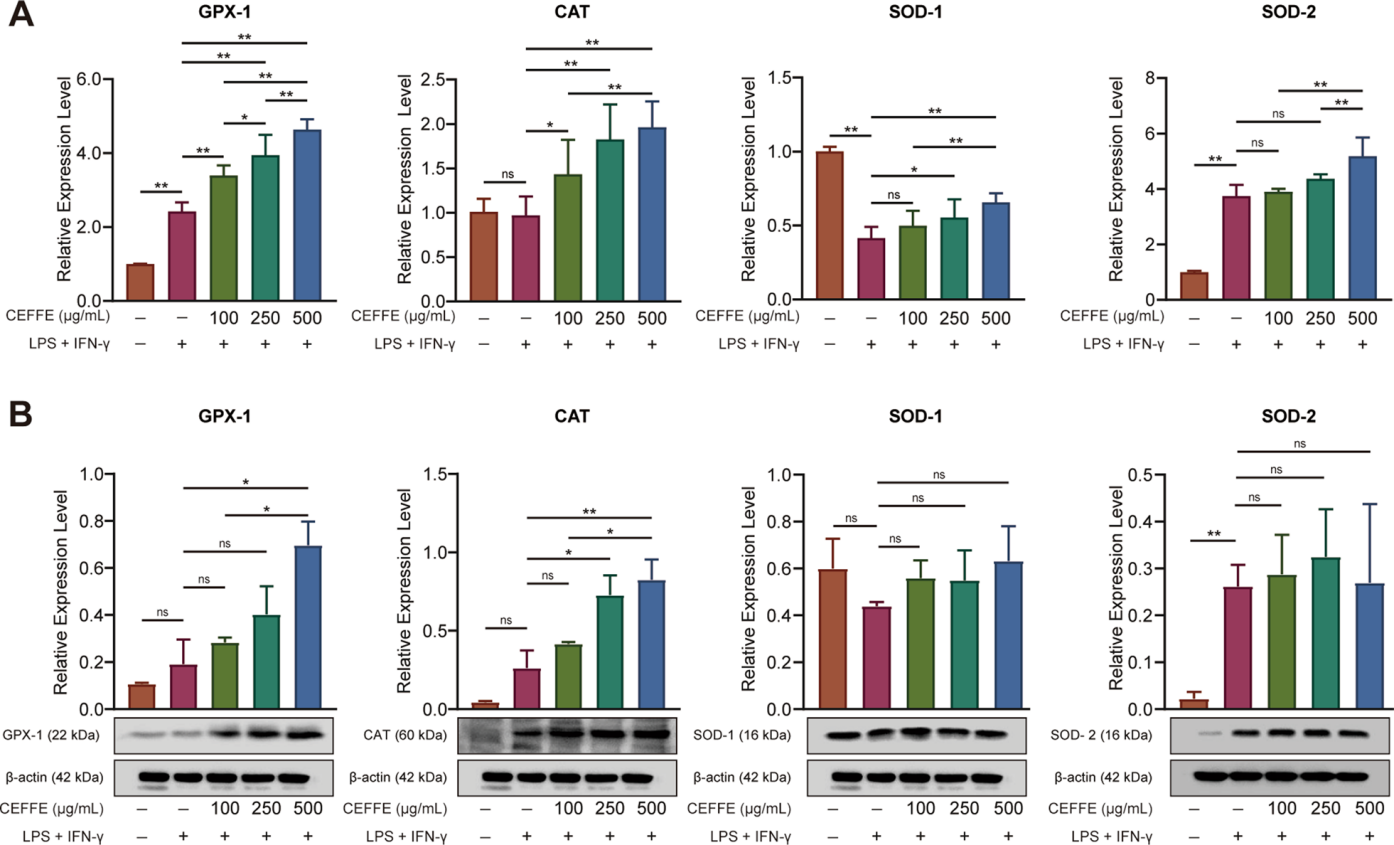
CEFFE promoted the expression of antioxidant enzymes in Raw 264.7 cells. **A** qRT-PCR analysis showed that the expression of antioxidant enzymes (GPX-1, CAT, SOD-1, and SOD-2) was increased after co-incubation with CEFFE. **B** Western blotting revealed an increase in the expression of antioxidant enzymes (GPX-1 and CAT) after co-incubation with CEFFE. GPX-1, CAT, and SOD-1 were from the same gel and SOD-2 was from another gel. Please refer to Additional file 1: Fig. S4 for original images. Data represent the mean ± SD (*n* = 3 per group). *p < 0.05; **p < 0.01; ns, no significant difference between groups

In addition, the expression of GPX-1, catalase, SOD-1, and SOD-2 antioxidant enzymes was increased as measured by qRT-PCR (Fig. 7A). Interestingly, the most apparent change was noticed for the expression of GPX-1 and catalase detected by western blot. (Fig. 7B).

**Fig. 8:**
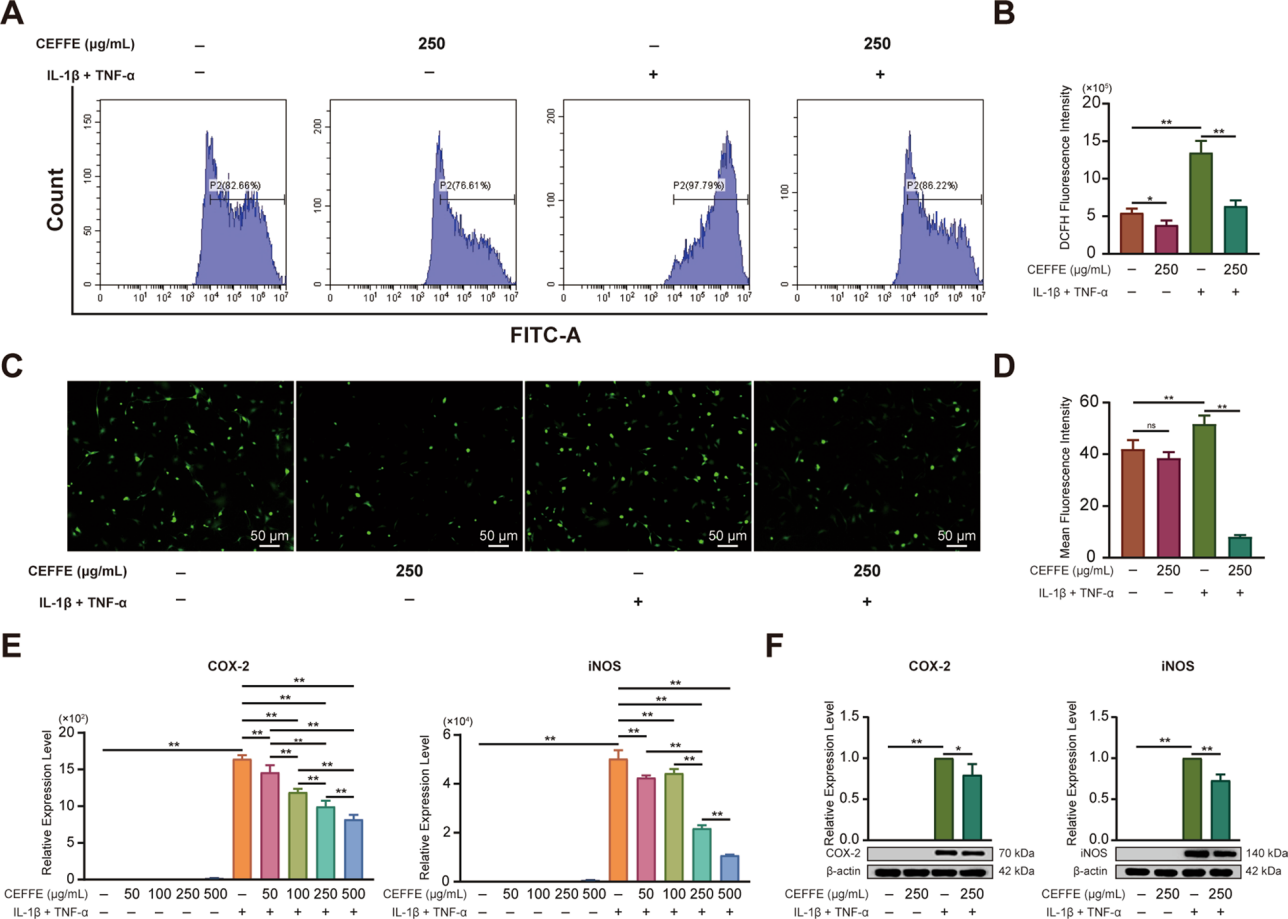
CEFFE reduced the intracellular production of ROS and expression of oxidative stress-related enzymes in murine primary chondrocytes. **A**, **B** Flow cytometry analysis revealed that CEFFE counteracted the intracellular production of ROS induced by IL-1β + TNF-α. **C**, **D** Fluorescence microscopy observation of DCHF fluorescence intensity showed a similar trend to that of flow cytometry. **E** qRT-PCR analysis showed that CEFFE did not affect the expression of oxidative stress-related enzymes (COX-2 and iNOS) in the absence of IL-1β + TNF-α but decreased their IL-1β + TNF-α-induced expression in a dose-dependent manner. **F** qRT-PCR analysis showed that CEFFE had no effect on the expression of oxidative stress-related enzymes (COX-2 and iNOS) in the absence of IL-1β + TNF-α, but decreased their IL-1β + TNF-α-induced expression. COX-2 and iNOS were from different gels. Please refer to Additional file 1: Fig. S5 for original images. Scale bars are noted on the right bottom corner of each picture. Data represent the mean ± SD (*n* = 3 per group). *p < 0.05; **p < 0.01; ns, no significant difference between groups

We also found that the fluorescence intensity declined in murine primary chondrocytes treated with 250 µg/mL CEFFE; in particular, CEFFE reduced the inflammation-induced fluorescence intensity of primary chondrocytes (Fig. 8A, B, C, D). We also detected the expression of COX-2 and iNOS using qRT-PCR and western blotting. CEFFE reduced the expression of both inflammation-activated COX-2 and iNOS (Fig. 8E, F).

## Discussion

At present, the clinical treatment of OA includes mainly non-pharmacological treatments through mechanical stimulation and symptomatic treatments, which mainly focus on pain management and do not facilitate the regeneration of cartilage or reduce joint inflammation [7, 18]. These limitations require the development of novel, simple, efficient, and sound therapeutic approaches. Many clinical trials or extensive animal experiments have reported that stem cells represent a valid candidate for the cure of OA due to their capacity to differentiate and their ability to produce paracrine growth factors, such as TGF-β1 and VEGF [23–26, 44, 45]. Moreover, some studies have demonstrated that an innovative cell-free strategy is equivalent to stem cell therapy, avoiding the disadvantages and ethical concerns associated with the use of stem cells [46]. In the current study, we demonstrated that CEFFE, which contained cytokines similar to those produced by stem cells, could promote cartilage regeneration and alleviate low-grade inflammation in an MIA-induced OA rat model. In addition, we explored the underlying mechanisms and confirmed the therapeutic effects of CEFFE in Raw 264.7 macrophages and murine primary chondrocytes.

It has been shown that inflammatory changes, especially the polarized phenotype of macrophages in OA synovium, correlate with the pathogenesis and progression of OA [47]. Stem cells, exosomes, and platelet-rich plasma (PRP) therapies can shift pro-inflammatory M1 into anti-inflammatory M2 macrophages by immunomodulatory bioactive factors through a series of complex mechanisms [48–50]. Our previous studies showed that CEFFE regulated inflammation by reducing the infiltration of macrophages in the skin [35]. In the current study, we confirmed the effectiveness of CEFFE in regulating macrophages both in vivo and in vitro*.* Using in vivo experiments, we found that CEFFE increased the proportion of anti-inflammatory M2 macrophages in the synovium, while through in vitro experiments, we found that CEFFE mediated inflammation by inhibiting the M0 to M1 polarization of macrophages. Concomitantly, we found that CEFFE reduced the expression of the IL-1β, IL-6, iNOS, and TNF-α pro-inflammatory factors, but increased the expression of the ARG and IL-10 anti-inflammatory factors. We speculated that multiple growth factors such as TFG-β and IGF-1in CEEFE might be responsible for the anti-inflammatory effects [31]. For instance, TGF-β might inhibit natural killer lymphocytes and the maturation of inflammatory macrophages [46]. IGF-1 might inhibit the activation of NF-κB and its downstream targets involved in inflammation, while hepatocyte growth factor might also reduce NF-κB signaling [51]. Therefore, we assumed that multiple components in CEFFE potentially function either independently or cooperatively [51].

The increased levels of ROS, which lead to elevated oxidative stress and direct damage to DNA, lipids, and proteins, also play an important role in the development of OA [52–54]. Furthermore, ROS also plays essential signaling functions in pathways including MAPKs and the NF-κB pro-inflammatory transcription factor, resulting in the increased expression of inflammatory cytokines, such as TNF-α, IL-1β, IL-6, and NO [55, 56]. Accumulating evidence suggests that the elimination of excess ROS facilitates cartilage reconstruction and OA recovery [57]. Our previous studies have suggested that CEFFE increases the expression of antioxidant enzymes in fibroblasts, showing characteristics of an antioxidant agent [34]. In this study, we confirmed the antioxidant stress ability of CEFFE in the macrophage and chondrocyte models in vitro. In RAW264.7 cells, CEFFE mainly reduced ROS by increasing the expression of antioxidant stress kinases, whereas, in chondrocytes, CEFFE exerted its effect by reducing the expression of the oxidative stress related enzymes.

Another essential feature of OA is cartilage destruction under the inflammatory state. Type II collagen is the main protein component of cartilage, forming a mesh-like structure to embed aggrecan and other proteoglycans [9, 58, 59]. Long-term cartilage degradation leads to the activation of chondrocytes, which is characterized by the production of inflammatory cytokines, such as IL-1β, TNF-α, and matrix-degrading enzymes including the metalloproteinase (MMP) and ADAMTs-5 [60]. Research focusing on stem cells and their derivatives for cartilage regeneration and reduction in the levels of ADAMTs-5 has shown promising results. Our previous studies indicated that CEFFE contains multiple growth factors that promote the proliferation and regeneration of fibroblasts and epidermal cells [32, 33]. The current study confirmed the ability of CEFFE to directly promote cartilage regeneration and reduce the level of matrix metalloproteinase and inflammatory mediators in vitro and *vivo*. Among those cytokines, TGF-β might be a crucial factor stimulating cartilage regeneration, as it promotes the expression of SOX-9 and type II collagen [46].

Same as we reported in our previous studies, the pro-angiogenic effect of CEFFE was also observed in this study (Additional file 1: Fig. S2). It has been reported that angiogenesis is not good for osteoarthritis [61]. However, MSC or platelet-rich plasma (PRP) also possesses pro-angiogenic activity but showed therapeutic effects in the treatment of osteoarthritis [62]. Whether the angiogenic property of CEFFE, MSC and PRP play a positive or negative role in osteoarthritis is not clear. It is worth to be investigated in future.

Studies have proved the effects of MSCs therapy in the treatment of osteoarthritis. The underline mechanism is likely related to the paracrine effects of MSCs. Thus, the supernatant and exosomes derived from cultured MSCs have also shown their effects in the treatment of osteoarthritis. Compared with MSC therapy, CEFFE is a cell-free liquid that could avoid the safety issues associated with cell-based therapies. Moreover, CEFFE was non-immunogenic and non-tumorigenicity, which could be obtained or used not only for autologous sources but also allogeneic ones potentially. Compared with the supernatant or exosome, CEFFE could be easily prepared in the operating room without cell culture. In addition, CEFFE could be stored at − 80 °C immediately after production without cryoprotectant and thawed once at the time of use [63].

The processing of fat tissue and isolation of CEFFE is simple and can be easily conducted in a clinical setup. Generally, CEFFE could be easily obtained due to the abundant liposuction waste and the high output at approximately 10–15% of fresh lipoaspirate. As for the clinical use policy, CEFFE is not considered as human cells, tissues, and cellular and tissue-based products (HCT/Ps) according to Food and Drug Administration (FDA), which make it easier to promote in clinical application [64, 65]. Meanwhile, in our current clinical prospective study targeting autologous CEFFE in the treatment of knee OA (Registration number: ChiCTR2100051039), the preparation process did not use any exogenous substances such as enzymes and could be considered within the minimally manipulated biological product category [66]. Specially, we have consulted some PRP clinical trials [67] and determined that participants would receive 3 intra-articular injections (1–2 mL/each; at weekly intervals) and 6-month follow-up. The required fat for a course of treatment was only 100–200 mL, which could achieve 15–30 mL of CEFFE. Following liposuction, routine clinical care including wound nursing, postural nursing, and prolonged use of elastic compression garments is required. Thus, it is more practicable in the clinic than stem cell-based therapies.

However, as the specific components of CEFFE remain incompletely defined, future investigation on quality control of CEFFE from different donors or different batches is required. For example, it has been reported that brain-derived neurotrophic factor (BDNF) and glial cell line-derived neurotrophic factor (GDNF) were positively associated with pain in OA [68, 69]. When BDNF was injected into the knees of rats with experimental OA induced with MIA, it worsened weight-bearing deficits and mechanical allodynia in the hind paw [69]. However, the amount of BDNF used in their study ranged from 0.1 to 10 µg (in 50 µL), which was much higher than the BDNF content (approximately 0.00012 µg in 60 µL) used in this study. Similarly, GDNF was 0.2 µg (in 10 µL) in [70], which was much higher than the GDNF content (approximately 0.00012 µg in 60 µL) used in this study. Moreover, OA symptoms were relieved after CEFFE treatment, indicating that it did not aggravate OA pain. Whether it is necessary to remove BDNF and GDNF from CEFFE warrants further evaluation in clinical trials. In the future, the use of purified functional proteins from CEFFE mixtures might take a more prominent role in treating OA.

In summary, by combining its excellent immunomodulation properties in regulating macrophage polarization and eliminating excess oxidative stress and cartilage protective effects, CEFFE appears to have promising therapeutic potential in OA.

## Conclusion

In conclusion, our results demonstrated that CEFFE could be used as a promising strategy to inhibit or delay the progression of early-stage OA by promoting cartilage regeneration and limiting low-grade joint inflammation.

## Supplementary Information

**Additional file 1: Figure S1**. The isolated primary chondrocytes confirmation.** Figure S2**. Capillary density in synovium.** Figure S3**. CEFFE did not show any effect on Raw 264.7 cells polarization without LPS + IFN-γ.** Figure S4**. The original data of SOD-2 western blot results and original images of western blot presented in Fig. 7B.** Figure S5**. The original data of COX-2 and iNOS western blot results and original images of western blot presented in Fig. 8F.

**Additional file 2: Table S1**. Gene primers used in the article.

## Data Availability

The datasets during the current study are available from the corresponding author on reasonable request.

## Abbreviations

ADAMTs-5: A disintegrin metalloproteinase with thrombospondin motifs 5
ADSCs: Adipose-derived stem cells
ARG: Arginase; BDNF: Brain-derived neurotrophic factor
CAT: Catalases
CD206: Mannose receptor
CEFFE: Cell-free fat extract
DCFH-DA: 2, 7-Dichlorodihydrofluorescein diacetate
DMEM: Dulbecco's modified Eagle medium
FBS: Fetal bovine serum
GAG: Glycosaminoglycan
GPX: Glutathione peroxidase
GDNF: Glial cell line-derived neurotrophic factor
HE: Hematoxylin–eosin
IFN: Interferon
IGF: Insulin-like factor
IL: Interleukin
iNOS: Inducible nitric oxide synthase
LPS: Lipopolysaccharide
MIA: Sodium iodoacetate
MMP: Metalloproteinase
MSCs: Mesenchymal stem cells
OA: Osteoarthritis
OARSI: Osteoarthritis Research Society International
PBS: Phosphate-buffered saline
qRT-PCR: Quantitative real-time polymerase chain reaction
ROS: Reactive oxygen species
S&F: Safranin O-fast green
SD: Sprague–Dawley
SOD: Superoxide dismutase
TGF: Transforming growth factor
TNF: Tumor necrosis factor
VEGF: Vascular endothelial growth factor
