# Supplementary material for: Cell-free fat extract attenuates osteoarthritis via chondrocytes regeneration and macrophages immunomodulation
Source: Stem Cell Res Ther. 2022 Apr 1;13:133. doi: 10.1186/s13287-022-02813-3 (PMC8973552; doi:10.1186/s13287-022-02813-3)
Supplement: Supplementary file 1 — Additional file 1: Figure S1. The isolated primary chondrocytes confirmation. Figure S2. Capillary density in synovium. Figure S3. CEFFE did not show any effect on Raw 264.7 cells polarization without LPS + IFN-γ. Figure S4. The original data of SOD-2 western blot results and original images of western blot presented in Fig. 7B. Figure S5. The original data of COX-2 and iNOS western blot results and original images of western blot presented in Fig. 8F. [file 13287_2022_2813_MOESM1_ESM.docx]

**Cell-Free Fat Extract Attenuates Osteoarthritis via Chondrocytes Regeneration and Macrophages Immunomodulation**

Zhuoxuan Jia^†^, Bijun Kang^†^, Yizuo Cai, Chingyu Chen, Zheyuan Yu^*^, Wei Li^*^, and Wenjie Zhang^*^

Department of Plastic and Reconstructive Surgery, Shanghai 9th People's Hospital, Shanghai Jiao Tong University School of Medicine, Shanghai Key Laboratory of Tissue Engineering, National Tissue Engineering Center of China, 639 ZhiZaoJu Road, Shanghai 200011, China

^*^ Correspondence: [zheyuan_yu@qq.com](mailto:zheyuan_yu@qq.com) (Zheyuan Yu); [liweiboshi@163.com](mailto:liweiboshi@163.com) (Wei Li); [wenjieboshi@aliyun.com](mailto:wenjieboshi@aliyun.com) (Wenjie Zhang)

^†^ Zhuoxuan Jia and Bijun Kang contributed equally to this work.

**
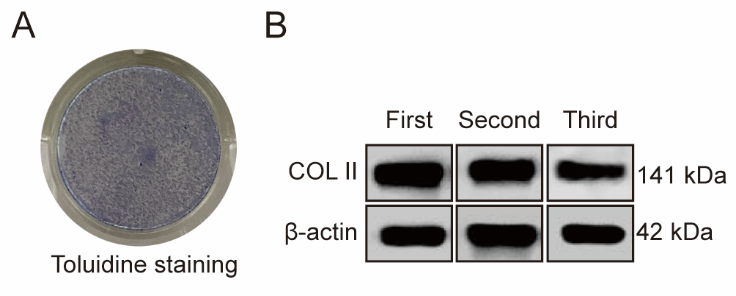
**

**Fig. S1.** The isolated primary chondrocytes confirmation. **A** Toluidine staining. **B** Western blot results.

**
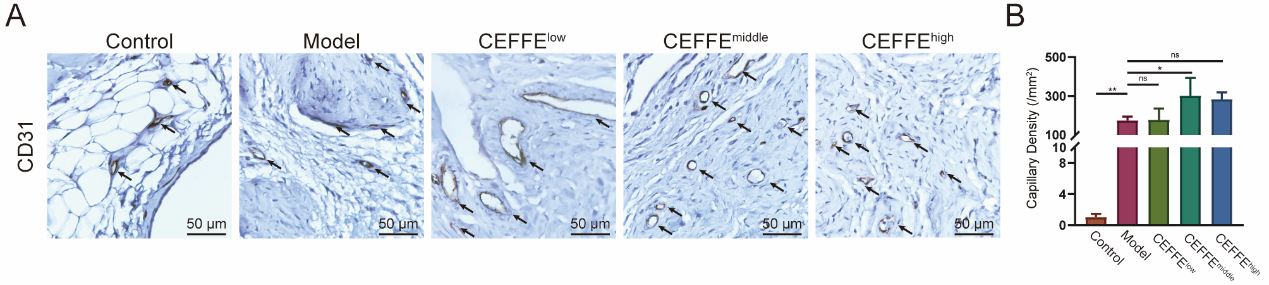
**

**Fig. S2.** Capillary density in synovium. **A** Anti-CD31 staining of knee synovium. **B** Qualification of capillary density. Scale bars are noted on the right bottom corner of each picture. Data represent the mean ± SD (n = 6 pes1 group). *p < 0.05; **p < 0.01; ns, no significant difference between groups.

**
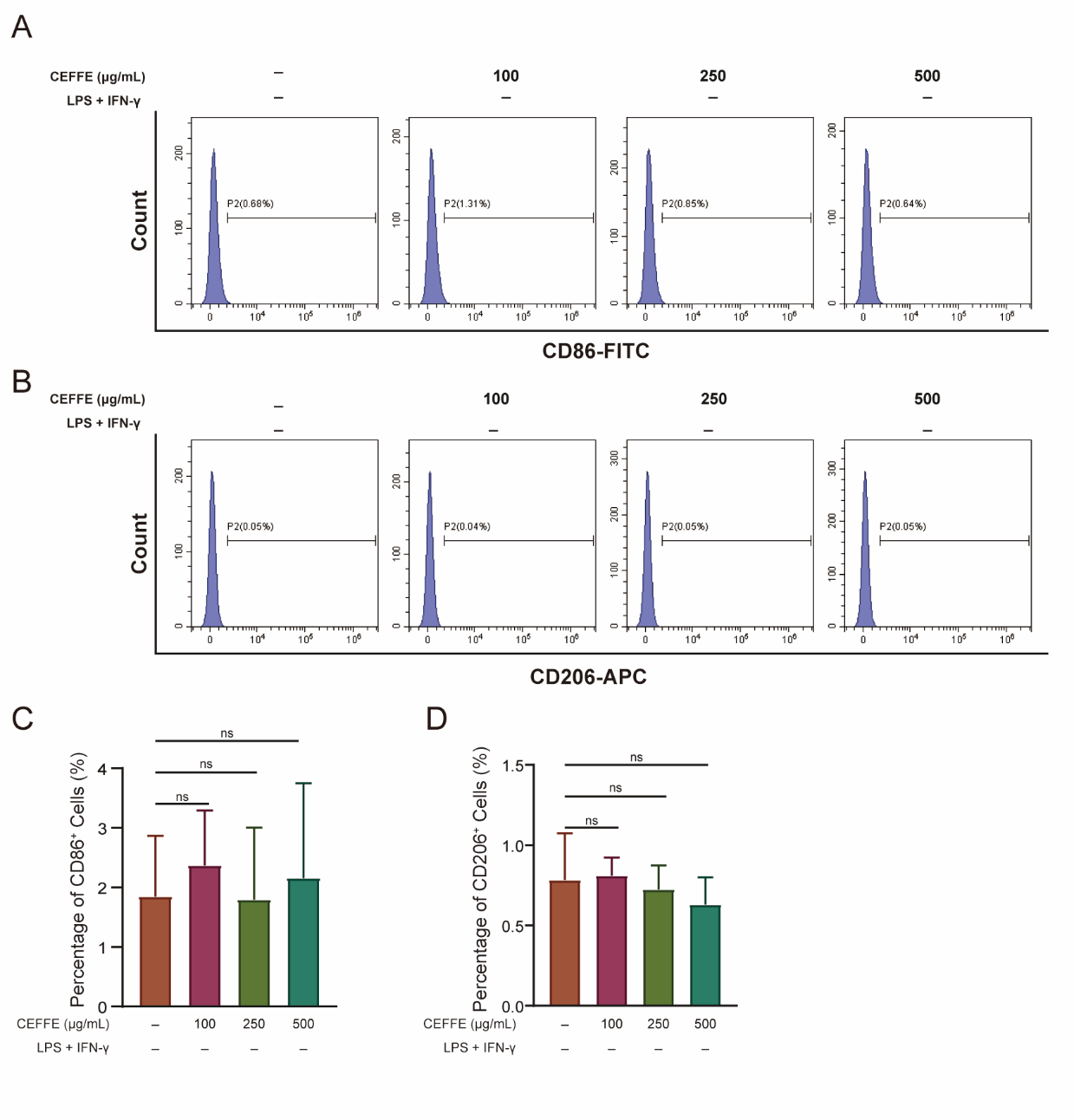
**

**Fig. S3.** CEFFE did not show any effect on Raw 264.7 cells polarization without LPS+IFN-γ. **A, C** Quantification of CD86-positive cells by flow cytometry. No obvious change in the proportion of M1 macrophages was observed after treatment with CEFFE. **B, D** Quantification of CD206 positive cells by flow cytometry. No obvious change was observed in the ratio of M2 cells. Data represent the mean ± standard deviation (n = 3 per group). ns, no significant difference between groups.

**
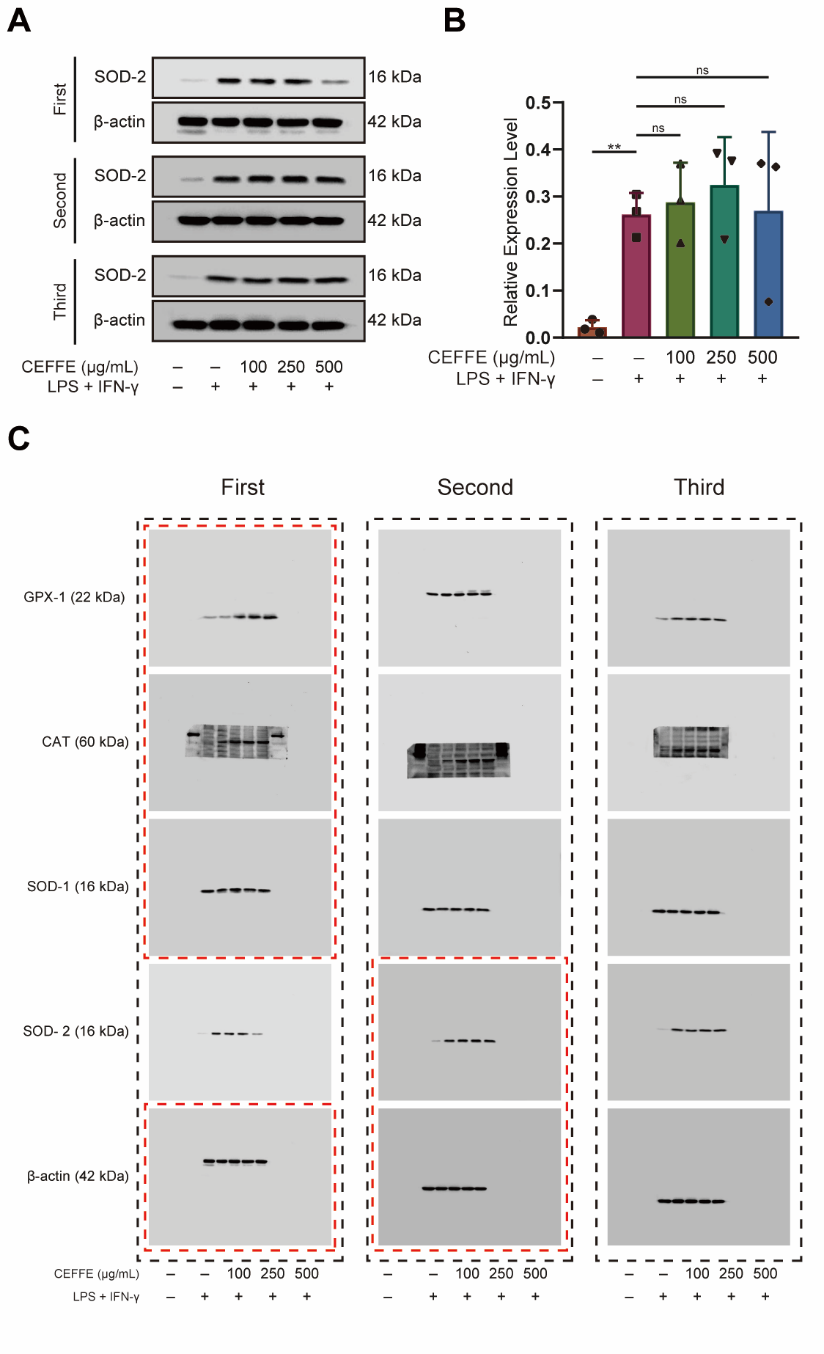
**

**Fig. S4.** **A, B** The original data of SOD-2 western blot results. **C** Original images of western blot presented in Fig. 7B. Images from the same gel was put in the black dashed box. Images presented in the manuscript was put in the red dashed box.


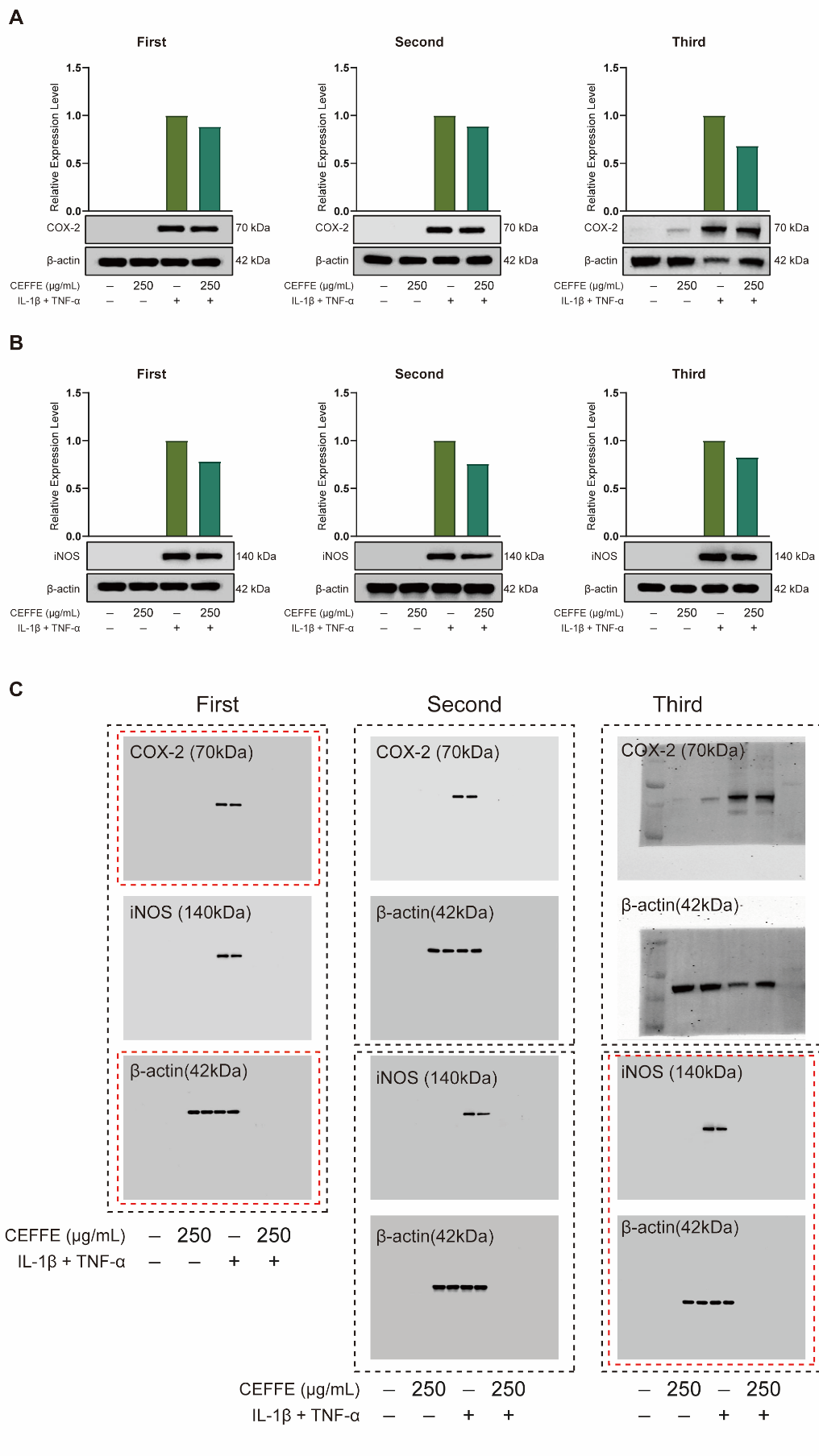


**Fig. S5.** **A, B** The original data of COX-2 and iNOS western blot results. **C** Original images of western blot presented in Fig. 8F. Images from the same gel was put in the black dashed box. Images presented in the manuscript was put in the red dashed box.
