# Supplementary material for: Cell-free fat extract attenuates osteoarthritis via chondrocytes regeneration and macrophages immunomodulation
Source: Stem Cell Res Ther. 2022 Apr 1;13:133. doi: 10.1186/s13287-022-02813-3 (PMC8973552; doi:10.1186/s13287-022-02813-3)
Supplement: Supplementary file 2 — Additional file 2: Table S1. Gene primers used in the article. [file 13287_2022_2813_MOESM2_ESM.docx]

| Mouse IL-1β-F | GAAATGCCACCTTTTGACAGTG |
| --- | --- |
| Mouse IL-1β-R | TGGATGCTCTCATCAGGACAG |
| Mouse IL-6-F | CTGCAAGAGACTTCCATCCAG |
| Mouse IL-6-R | AGTGGTATAGACAGGTCTGTTGG |
| Mouse iNOS-F | ACATCGACCCGTCCACAGTAT |
| Mouse iNOS-R | CAGAGGGGTAGGCTTGTCTC |
| Mouse TNF-α-F | CAGGCGGTGCCTATGTCTC |
| Mouse TNF-α-R | CGATCACCCCGAAGTTCAGTAG |
| Mouse IL-10-F | GCCCTTTGCTATGGTGTC |
| Mouse IL-10-R | TCTCCCTGGTTTCTCTTCC |
| Mouse ARG-F | CTCCAAGCCAAAGTCCTTAGAG |
| Mouse ARG-R | GGAGCTGTCATTAGGGACATCA |
| Mouse TGF-β-F | CCAGATCCTGTCCAAACTAAGG |
| Mouse TGF-β-R | CTCTTTAGCATAGTAGTCCGCT |
| Mouse CD206-F | TGGAGGCTGATTACGAGCAGT |
| Mouse CD206-R | TTGGTTCACCGTAAGCCCAAT |
| Mouse GPX-1-F | GTTTGAGAAGTGCGAAGTGAAT |
| Mouse GPX-1-R | CGGAGACCAAATGATGTACTTG |
| Mouse CAT-F | CACCTTCAAGTTGGTTAATGCA |
| Mouse CAT-R | CATGACCTGGATGTAAAACGTC |
| Mouse Sod-1-F | TGTCCATTGAAGATCGTGTGAT |
| Mouse Sod-1-R | TCATCTTGTTTCTCATGGACCA |
| Mouse Sod-2-F | AAGGGAGATGTTACAACTCAGG |
| Mouse Sod-2-R | GCTCAGGTTTGTCCAGAAAATG |
| Mouse GAPDH-F | ATGGTGAAGGTCGGTGTGAA |
| Mouse GAPDH-R | TGAGTGGAGTCATACTGGAACA |
| Mouse ADAMTS-5-F | AATGGGTTCCCAAATATGCAGGTGT |
| Mouse ADAMTS-5-R | GTCCCATCCGTAACCTTTGGAGA |
| Mouse SOX-9-F | TATCTTCAAGGCGCTGCAA |
| Mouse SOX-9-R | TCGGTTTTGGGAGTGGTG |
| Mouse COX-2-F | AACCTGCCTAGCAGAACGAC |
| Mouse COX-2-R | GCGCGCTCCTAGTACTCAAT |

Table 1 Gene primers used in the article.
